# Antimetastatic Potentials of *Dioscorea nipponica* on Melanoma *In Vitro* and *In Vivo*

**DOI:** 10.1155/2011/507920

**Published:** 2011-01-10

**Authors:** Mao-Lin Ho, Yih-Shou Hsieh, Jia-Yuh Chen, Kuo-Shuen Chen, Jia-Jing Chen, Wu-Hsien Kuo, Shu-Jiuan Lin, Pei-Ni Chen

**Affiliations:** ^1^Institute of Medicine, Chung Shan Medical University, No. 110, Section 1, Jianguo N. Road, Taichung 402, Taiwan; ^2^Department of Biochemistry, Chung Shan Medical University, No. 110, Section 1, Jianguo N. Road, Taichung 402, Taiwan; ^3^Clinical Laboratory, Chung Shan Medical University Hospital, No. 110, Section 1, Jianguo N. Road, Taichung 402, Taiwan; ^4^Department of Internal Medicine, Chung Shan Medical University Hospital, No. 110, Section 1, Jianguo N. Road, Taichung 402, Taiwan; ^5^Institute of Biochemistry and Biotechnology, Chung Shan Medical University, No. 110, Section 1, Jianguo N. Road, Taichung 402, Taiwan; ^6^Division of Gastroenterology, Department of Internal Medicine, Armed-Force Taichung General Hospital, Taichung 411, Taiwan; ^7^General Education Center, Central Taiwan University of Science and Technology, No. 11 Pu-tzu Lane, Pu-tzu Road, Taichung 406, Taiwan; ^8^Department of Pathology, Taichung Veterans General Hospital, Taichung 407, Taiwan

## Abstract

Recent studies have revealed pleiotropic anticancer and antiproliferative capabilities of *Dioscorea nipponica* Makino whereas the effect of this plant on metastasis of cancer cells has not been clearly clarified. In the present study, we extracted *Dioscorea nipponica* Makino with methanol (DNE1), chloroform (DNE2), ethyl acetate (DNE3), *n*-butanol (DNE4), and water (DNE5). We first demonstrate that DNE3 was found to be effective in reducing the lung metastases formation by about 99.5% as compared to vehicle-treated control animals. When a nontoxic concentration of the extract was treated directly to highly metastatic murin melanoma cells (B16F10) and human melanoma cells (A2058) *in vitro*, it exerted a dose-dependent inhibitory effect on the invasion (*P* < .001), motility (*P* < .001), secretion of MMPs (*P* < .001), and u-PA (*P* < .001) of both cell lines. To investigate the possible mechanisms involved in these events, we performed western blot analysis to find that DNE inhibited phosphorylation of Akt. A treatment with DNE3 to B16F10 cells also inhibited the activation of NF-*κ*B and increased the expression of IkappaB. Taken together, these findings suggested that DNE3 could reduce the metastasis of melanoma cells, thereby constituting an adjuvant treatment for metastasis control.

## 1. Introduction

Epidemiological studies have shown that a diet rich in vegetables and fruits might protect against cancer by mechanisms that have not been well defined yet. In recent years, naturally occurring plant products have been getting increased attention for the intervention of malignant invasive progression in the late stage of neoplastic diseases [1, 2]. On the basis of this idea, certain foods, including many vegetables, fruits, and grains, as well as phytochemicals of diversified pharmacological efficacies have been shown to offer a significant protection against various cancers [3–5]. Furthermore, there is an increase focus on providing scientific basis to use these agents in the prevention strategy for people with a high risk for cancers. *Dioscorea nipponica* Makino has long been used as a folk medicine for bronchitis, asthma, and rheumatoid arthritis. These compounds were effective for preventing both the body and adipose tissue weight gains in rodents induced by a high-fat diet [6]. However, limited studies are available concerning the effect of *Dioscorea nipponica* Makino in anticancer. 

Invasion and metastasis of solid tumor involves multiple processes and various cytophysiological changes, including changed adhesion capability between cells and extracellular matrix (ECM) and damaged intercellular interaction [7]. Degradation of ECM by cancer cells via protease, such as metalloproteinases (MMPs), serine proteinases, plasminogen activator (PA), and cathepsins, may lead to the separation of intercellular matrix to promote the motility of cancer cells and eventually lead to invasion and metastasis. Among these peoteinase, MMP-2 and MMP-9 are type IV collagenases that degrade basement membrane collagen [8]. These two MMPs are expressed in many different types of cancer cells; however, they are predominately produced in stromal cells located adjacent to the tumors [9]. In human malignancies, increased MMP-2 and MMP-9 activity and expression correlate with reduced survival and poor disease prognosis [10–13]. 

However, these studies on functions of *Dioscorea nipponica* Makino have been mainly focused on the effects of antiallergic property or antiobesity whereas the effect of this plant on migration and invasion of tumor cells has not been clearly clarified. The purpose of the present study was to characterize the effects of *Dioscorea nipponica* Makino on tumor cell metastasis *in vivo*, and cell invasion, migration, motility, adhesion, and proteinase expression *in vitro*.

## 2. Methods

### 2.1. Chemicals

*Dioscorea nipponica* Makino was obtained from a plantation of the Green Health Biotechnology Corporation (Yunlin, Taiwan) and identified by Professor Yih-Shou Hsieh, Chung Shan Medical University. 3-(4,5-dimethylthiazol-2-y1)-2,5-diphenyltetrazolium bromide (MTT) and Dulbecco's modified Eagle medium (DMEM) were obtained from Sigma Chemical Co. (St. Louis, MO, USA) and Matrigel was purchased from BD Biosciences (Bedford, MA, USA). Rabbit polyclonal antibodies against c-Jun and c-Fos were purchased from Biosource (Camarillo, CA, USA) and a rabbit polyclonal antibody against tissue inhibitor of matrix metalloproteinase-2 (TIMP-2) was purchased from Serotec (Oxford, UK). Monoclonal antibodies against nuclear factor-*κ*B (NF-*κ*B) and C23 and rabbit polyclonal antibodies against Extracellular signal-regulated kinase 1/2 (ERK1/2), p38, and Akt, the total and phosphorylated protein, were purchased from Santa Cruz Biotechnology Inc. (Santa Cruz, CA, USA). A monoclonal antibody against plasminogen activator inhibitor (PAI) was obtained from American Diagnostics Inc. (Greenwich, CT, USA). ECL Plus detection kit was obtained from Amersham Life Sciences, Inc. (Piscataway, NJ). Lightshift kit was purchased from Pierce (Rockford, IL, USA).

### 2.2. Preparation of Dioscorea nipponica Makino Extracts

Air-dried *Dioscorea nipponica* Makino (50 g) was extracted 3 times with boiling water (500 mL) for 30 minutes, and the filtrate was partitioned with chloroform (DNE2), ethyl acetate (DNE3), and *n*-butanol (DNE4), and the residue was extracted with methanol (DNE1). Afterwards, solvent was removed by a vacuum rotary evaporator and these fractions were lyophilized and stored at −20°C. Furthermore, the chemical profile of ethyl acetate soluble fraction was analyzed by using high-pressure liquid chromatograms- (HPLC-)-mass spectrometer. Briefly, DNE3 was analyzed by HPLC-mass spectrometer using a HPLC (Hitachi L-6200 with an L-4500 Diode Array detector) with a PE Sciex Qstar Pulsar ESI-TOF mass spectrometer. Samples (10 *μ*l) were injected into a Merck LiChrospher 100 RP-18 column (4 × 250 mm). The column was equilibrated in 0.05% acetic acid/water (solution A), and elution of the components was performed by increasing the concentration of acetonitrile (solution B) from 0% to 60% in 30 minutes at a flow rate of 1 mL/min. The main product peak with a retention time of 16.644 minutes was then subjected to electrospray ionisation mass spectra using multiply-charged ion profile based on the modified method of Wong et al. [14].

### 2.3. Cell Culture

Cell lines including A2058 from human melanoma, B16F10 from a mouse melanoma, and HS68 from a human foreskin fibroblast cells were obtained from American Type Culture Collection (Rockville, MD, USA) and cultured in DMEM (for A2058 and B16F10) or RPMI (for HS68).

### 2.4. Measurement of Lung Metastasis in B16F10-Bearing Mice

C57BL/6 male mice of 5-week-old (National Taiwan University Animal Center, Taiwan) were housed with a regular 12-hour light/12-hour dark cycle and *ad libitum* access to standard rodent chow diet (Laboratory Rodent Diet 5001, LabDiet, St. Louis, MO). Cells (2 × 10^5^ cells) suspended in 0.1 mL of PBS were injected into the tail vain of C57BL/6 mice. On the following day (Day 1), mice were randomly divided into three groups (*n* = 8 for each group) to be fed by oral gavage with saline (control) or DNE3 (0.1 g/kg and 0.2 g/kg of body weight, daily). Five untreated mice were used as wild type control. After 21 days, animals were euthanized with CO_2_. The lungs were isolated and weighed, and metastatic nodules on the surface of lungs were counted under a microscopy. Lungs were fixed in neutral buffered 5% formalin, and sections were taken and stained with hematoxyline and eosine for morphological studies [15].

### 2.5. Determination of Cell Viability (MTT Assay)

Cells were treated with DNE3 (0, 25, 50, 75, and 100 *μ*g/mL) for 24 hours. After treatment, cells were incubated with 0.5 mg/mL MTT in culture medium for an additional 4 h, and the blue formazan crystals of viable cells were dissolved by lysis buffer (isopropyl alcohol containing 10% Triton X-100 and 0.1 N HCl) and measured spectrophotometrically at 570 nm [4].

### 2.6. Transwell Cell Invasion and Motility Assays

After a pre-treatment with DNE3 for 24 hours, cells were harvested and seeded to BD Falcon cell culture inserts (BD Biosciences, Bedford, MA, USA) at 5 × 10^4^ cells/well in serum-free medium and then incubated for another 12 h at 37°C. For invasion assay, 100 *μ*l Matrigel (1 mg/1 mL) was applied to the membrane filters with a pore size of 8 *μ*m and the bottom chamber contained standard medium. Filters were then air-dried for 5 h in a laminar flow hood. The invaded cells were fixed with methanol and stained with Giemsa. Cell numbers were counted under a light microscope. The motility assay was carried out as described in the invasion assay with no coating of Matrigel [16].

### 2.7. Wound Healing Migration Assay

Wounds were introduced to the confluent monolayer of cells with a yellow plastic pipette tip to create a cleared line. The medium was removed and replaced with DMEM containing 1% FBS, and then DNE3 was added. The cells were incubated at 37°C, and cell movement into the wound area was photographed at 0 h, 24 hours and 48 hours using a microscope.

### 2.8. Cell-Matrix Adhesion Assay

After a 24-hour treatment with DNE3, cells were plated on 24-well dishes coated with 150 *μ*l type I collagen (10 *μ*g/mL) and cultured for 30 minutes. Nonadherent cells were removed by PBS washes and adherent cells were fixed in ethanol. After staining with 0.1% crystal violet, fixed cells were lysed in 0.2% Triton X-100 and the absorbance was measured at 550 nm [17].

### 2.9. Determination of MMP-2, -9, and u-PA

In gelatin zymography, collected media were prepared with sodium dodecyl sulphate (SDS) sample buffer without boiling or reduction and subjected to 0.1% gelatin-8% SDS polyacrylamide gel electrophoresis (SDS-PAGE) to determine the MMPs. After electrophoresis, gels were washed with 2.5% Triton X-100 and then incubated in reaction buffer (40 mM Tris-HCl, pH 8.0, 10 mM CaCl_2_, 0.01% NaN_3_) for 16 h at 37°C. Then gel was stained with Coomassie brilliant blue R-250 [17].

Visualization of u-PA activity was performed by casein zymography as previously described [17]. Briefly, 2% w/v casein and 20 *μ*g/mL plasminogen were added to 8% SDS-PAGE gel, and then performed as described in the gelatin zymography.

### 2.10. Activating Protein-1 (AP-1) and NF-*κ*B Binding Assay

Binding of AP-1 and NF-*κ*B in nuclear extracts was assessed by electrophoretic mobility shift assay (EMSA) with biotin-labeled double-stranded AP-1 or NF-*κ*B oligonucleotides, and EMSA was carried out by using the Lightshift kit. Briefly, binding reactions containing 10 *μ*g of nuclear protein, 10 mM Tris, 50 mM KCl, 1 mM DTT, 5 mM MgCl_2_, 2 *μ*g poly (dI · dC), and 2 pmol of oligonucleotide probe were incubated for 20 minutes. Specific binding was confirmed by using a 200-fold excess of unlabeled probe as specific competitor. Protein-DNA complexes were separated by using a 6% nondenaturing acrylamide gel electrophoresis and then transferred to positively charged nylon membranes and crosslinked in a Stratagene crosslinker. Gel shifts were visualized with a streptavidin-horseradish peroxidase followed by chemiluminescent detection [18].

### 2.11. Western Blot Analysis

Samples were separated in a 10% polyacrylamide gel and transferred onto a nitrocellulose membrane as previously described [17]. The blot was subsequently operated with standard procedures and probed with each specific antibody at 4°C overnight. The protein expression was detected by chemiluminescence using an ECL Plus detection kit and relative photographic density was quantitated by scanning the photographic negatives on a gel documentation and analysis system.

### 2.12. Statistical Analysis

Statistical significances were analyzed by one-way analysis of variance (ANOVA) with *post-hock* Dunnett's test. *P*-value ≤ .05 was considered statistically significant (Sigma-Stat 2.0, Jandel Scientific, San Rafael, CA, USA). 

## 3. Results

### 3.1. Effects of DNE on the Invasion of Human Melanoma Cells

**Figure 1:**
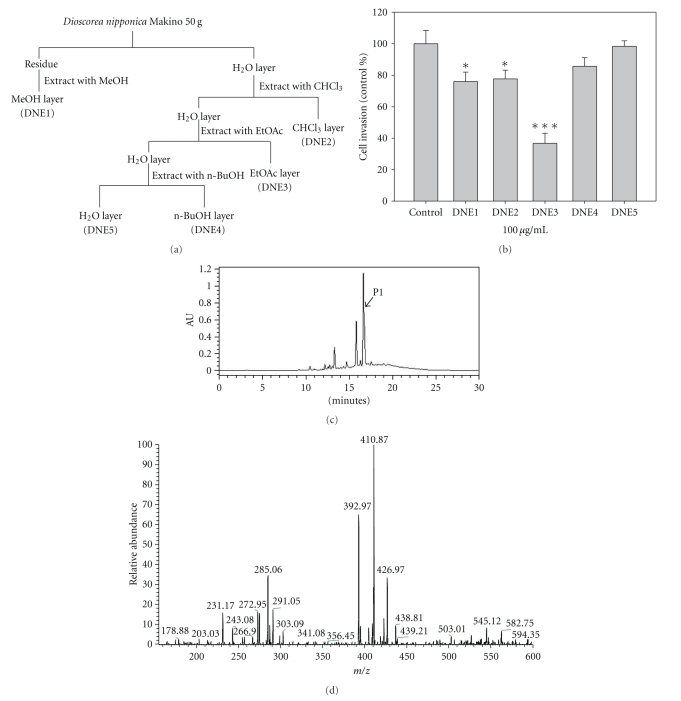
The chemical profile of *Dioscorea nipponica *extract was analyzed by HPLC-mass spectrometry. (a) Procedure for fractionation of the extracts from *Dioscorea nipponica.* (b) B16F10 cells were treated with these fractions by Transwell invasion assay. (c) Chromatographic patterns from HPLC analysis of DNE3 extracts showed peaks corresponding to the retention times (minutes). Absorbance was monitored at 254 nm. (d) The main product peak (P1) with a retention time of 16.644 minutes was then subjected to mass spectrometer.

To evaluate the bioactive compound of *Dioscorea nipponica* Makino, we successively extracted the *Dioscorea nipponica *with methanol (DNE1), chloroform (DNE2), ethyl acetate (DNE3), *n*-butanol (DNE4), and water (DNE5) (Figure 1(a)). B16F10 cells were treated with different *Dioscorea nipponica* extracts, and a decrease of invasion was detected by Transwell invasion assay. Among these extracts, DNE3 was the most effective *Dioscorea nipponica *ingredient that reduces invasion activity in our assay (Figure 1(b)). Chromatographic patterns from HPLC analysis of DNE3 extracts showed peaks corresponding to the retention times. Absorbance was monitored at 254 nm (Figure 1(c)). The main product peak (P1) with a retention time of 16.644 minutes as shown in Figure 1(c) was then subjected to electrospray ionisation mass spectra using multiply-charged ion profile (Figure 1(d)).

### 3.2. Inhibition of the Lung Colonization of B16F10 Melanoma by the Treatment of DNE3

**Figure 2:**
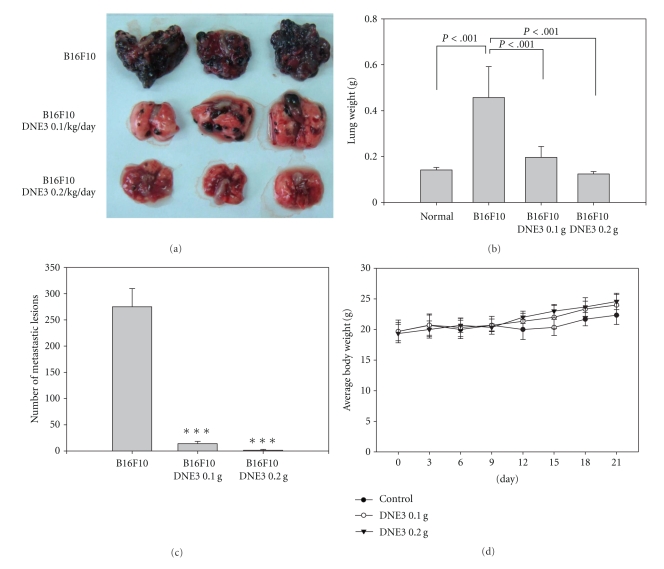
Suppression of lung metastasis of melanoma cells by DNE3. Melanoma cells were injected into the tail veins of 6-week-old male C57BL/6 mice. After injection of melanoma cells, DNE3 (0.1 g/kg/day and 0.2 g/kg/day) and vehicle (saline) alone were administered oral gavage for 21 days. Mice were sacrificed and then analyzed for representative photographs of lungs (a), the weight of lung (b), the number of lung metastasis (c), and the body weight of mice (d). Results were statistically evaluated by using one-way ANOVA with *post-hock* Dunnett's test (***P < .001).

**Figure 3:**
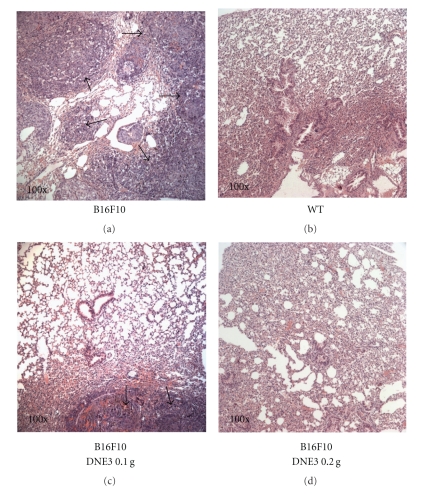
Histopathology of lung of metastatic tumor bearing animals (×100). Lungs of the metastasis-induced animals were fixed in neutral buffered formalin and stained with hematoxyline and eosine. (a) Control (melanoma + saline), (b) normal lung (wild type, WT), (c) melanoma + 0.1 g/kg/day DNE3, and (d) melanoma + 0.2 g/kg/day DNE3. Arrows showed areas of metastatic nodules (tumor).

Recent studies have shown that B16F10 cells mainly form lung tumors. C57BL/6 mice were injected via the tail vein with B16F10 melanoma cells, and administration of the ethyl acetate extracts of DEN3 reduced pulmonary metastasis formation of B16F10 cells. Within 21 days of injection, the control mice were visibly riddled with metastatic tumor nodules compared with the lungs of DNE3 treated mice (Figure 2(a)). Mean lung weights for animals receiving 0.1 g/kg/day DNE3 (0.1964 ± 0.0594 g; *P* < .001) and 0.2 g/kg/day DNE3 (0.1240 ± 0.0125 g; *P* < .001) were significantly lower than those from control animals (0.4570 ± 0.1488 g; Figure 2(b)). Vehicle-treated control animals had massive growth of tumor and was given an arbitrary-maximum countable number about 275 ± 45.3. It was reduced to 14 ± 5.4 (0.1 g/kg/day; *P* < .001) and 1.2 ± 1.7 (0.2 g/kg/day; *P* < .001) countable colonies by DNE3 treatment (Figure 2(c)). The average body weight of DNE3 treated mice was higher than control group (Figure 2(d)). Histopathology of the lung also showed marked reduction in tumor mass in the lungs of DNE3-treated animals (Figure 3).

### 3.3. Effect of DNE3 on Cell Viability

**Figure 4:**
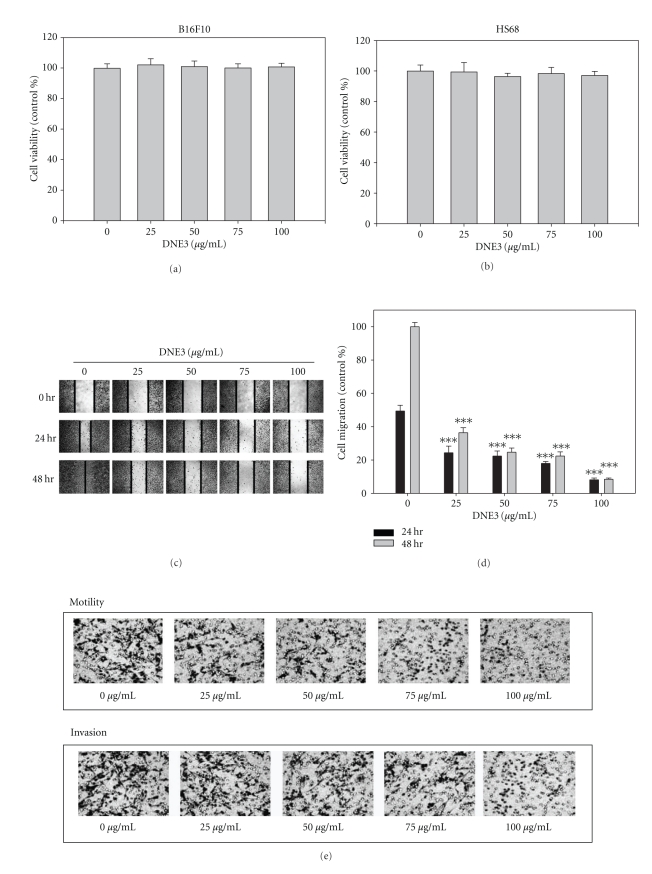

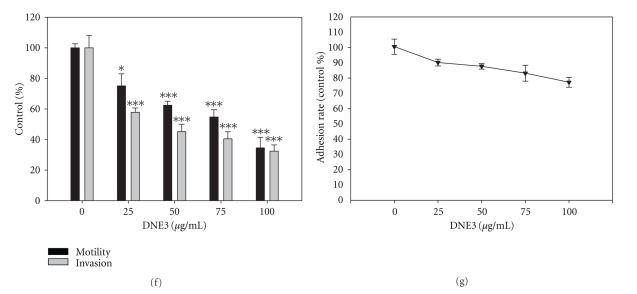
The effects of DNE3 on cell viability, migration, motility, invasion, and adhesion of melanoma cells. (a) B16F10 and (b) HS68 cells were treated with DNE3 for 24 hours by MTT assay. (c) B16F10 cells were subjected to analyze for cell migration by wound healing assay. (d) Determined migration ability of B16F10 was subsequently quantified with that of control being 100% (without DNE3 for 48 h). Cells were treated with DNE3 for 24 hours and then subjected to analyze for (e and f) motility, invasion, and (g) adhesion as described in Materials and Methods. Results were statistically evaluated by using one-way ANOVA with post hoc Dunnett's test (***P < .001). Results from 3 repeated and separated experiments were similar.

Results of MTT assay showed that DNE3 has no cytotoxicity on B16F10 cells (Figure 4(a)). Results from same procedures performed on normal human foreskin fibroblast cell lines, HS68, revealed that this compound did not have any significant cytotoxicity on these cells (Figure 4(b)).

### 3.4. Effects of DNE3 on the Invasion, Motility, Migration, and Adhesion

Incubation of B16F10 with 1% FBS produced a marked cell migration in the wound area 24 hours and 48 hours after wounding whereas wounds treated with DNE3 showed significant delays in wound healing under the same conditions (Figures 4(c) and 4(d)). To screen for the preventive effectors against cancer metastasis, the inhibitory effect of DNE3 on invasion and motility of B16F10 cells was examined with Transwell. The result showed that DNE3 significantly reduced the motility and invasion of B16F10 cells in a concentration-dependent manner (Figures 4(e) and 4(f)). 

Cancer cells, invading the host tissue, break from their cell-cell contacts and make new contact with the ECM. Therefore, DNE3 was tested to determine their effects on the cell-matrix adhesion. The results showed that DNE3 significantly reduced the cell-matrix interactions of B16F10 cells (Figure 4(g)).

### 3.5. Effects of DNE3 on the Activities of MMP-2, u-PA, and TIMP-2

**Figure 5:**
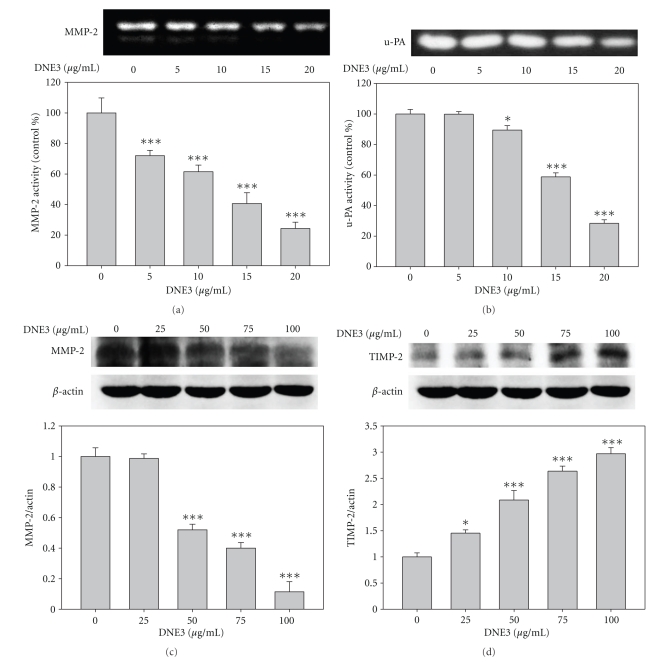
Effects of DNE3 on the protein levels of proteases and their endogenous inhibitors. Cells were treated with DNE3 for 24 hours and then condition media were subjected to gelatin zymography and casein zymography to analyze the activities of MMP-2 (a) and u-PA (b), respectively. Cell lysate were subjected to Western blot to analyze the expression of MMP-2 (c) and TIMP-2 (d). Results were statistically evaluated by using one-way ANOVA with post hoc Dunnett's test (*P < .05; ***P < .001).

Via gelatin and casein zymography assays, respectively, it has been shown that DNE3 significantly reduced MMP-2 (Figure 5(a)) and u-PA (Figure 5(b)) activity in dose-dependent manner. The immunoblotting showed that MMP-2 protein level was gradually decreased along with the concentration of DNE3 (Figure 5(c)). Physiological activity of MMP-2 is greatly related to that of their specific endogenous inhibitors, TIMP-2, therefore western blotting was employed to see the effect of DNE3 on TIMP-2 expression, and results showed that TIMP-2 protein levels were significantly increased along with the concentration of DNE3 in B16F10 (Figure 5(d)).

### 3.6. The Inhibition of Akt Phosphorylation by DNE3

**Figure 6:**
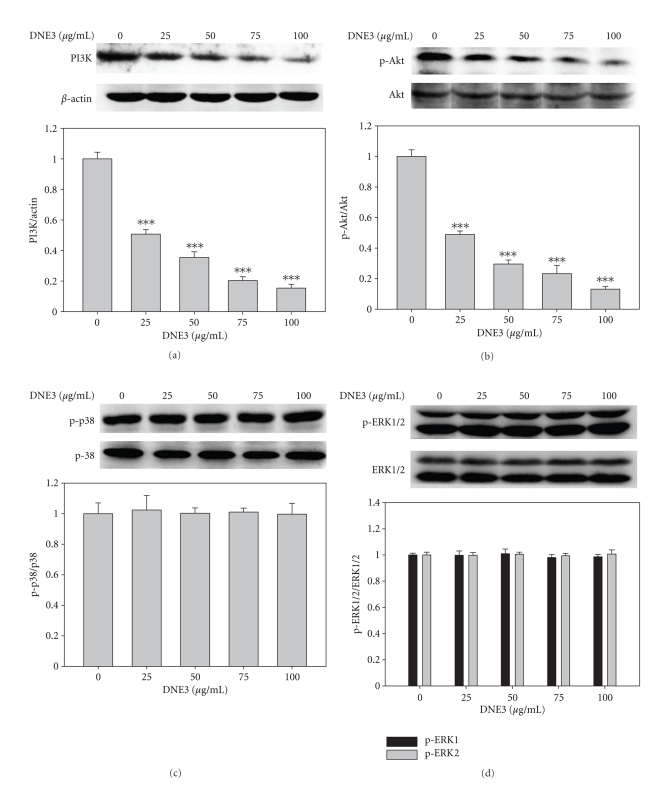
Inhibitory effect of DEN3 on the phosphorylation of Akt. B16F10 cells were cultured in various concentrations of DNE3 for 24 hours, and then cell lysates were subjected to SDS-PAGE followed by Western blotting with anti-PI3K (a), anti-phospho-Akt (b), anti-phospho-p38 (c), and anti-phospho-ERK1/2 (d) antibodies. Signals of proteins were visualized with an ECL detection system. Determined activities of these proteins were subsequently quantified by densitometric analysis with that of control being 100% (defined as 1.0) as shown just below the gel data. Results were statistically evaluated by using one-way ANOVA with post hoc Dunnett's test (***P < .001).

The immunoblotting showed that PI3K protein level was gradually decreased along with the concentration of DNE3 (Figure 6(a)). DNE3 significantly inhibited the activation of Akt as evidenced by a decrease in the levels of phospho-Akt proteins in a dose-dependent manner (Figure 6(b)) whereas it has no significant effect on p38 and ERK1/2 activity (Figures 6(c) and 6(d)).

### 3.7. Effects of DNE3 on the Activation of NF-*κ*B, c-Jun, and c-Fos

**Figure 7:**
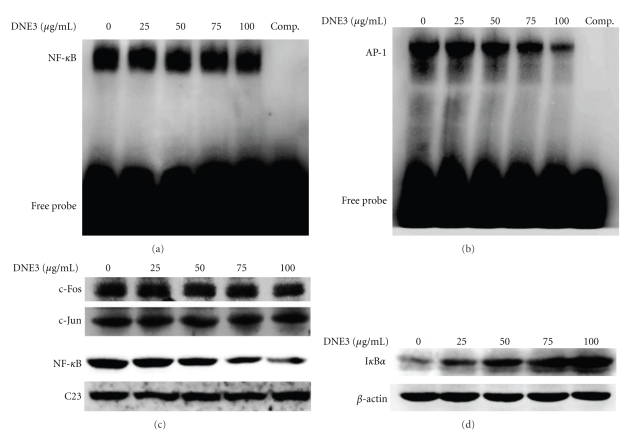
Effects of DNE3 on the activation of NF-*κ*B, c-Jun, and c-Fos. Cells were treated with DNE3 and then nuclear extracts were analysed for DNA binding activity of NF-*κ*B (a) and AP-1 (b) using biotin labeled NF-*κ*B and AP-1 specific oligonucleotide in EMSA. The last lane represented nuclear extracts incubated with unlabeled oligonucleotide (comp) to confirm the specificity of binding. Nuclear and cytoplasmic extracts were subjected to SDS-PAGE followed by western blotting with anti-NF-*κ*B, c-Fos, c-Jun, or C23 antibodies (c) and anti-IkB*α* or *β*-actin antibodies, respectively. Signals of proteins were visualized with an ECL detection system. The experiments were repeated three times with similar results.

To examine the effect of DNE3 on the activation of NF-*κ*B and AP-1, B16F10 cells were treated with DNE3 for 24 hours. Nuclear extract was analyzed by EMSA for AP-1 and NF-*κ*B DNA binding activity, and results showed that DNA binding activity of NF-*κ*B (Figure 7(a)) was significantly inhibited by a treatment with 100 *μ*g/mL DNE3 whereas the AP-1 activity was unchanged (Figure 7(b)). Subsequently, western blot was performed to further confirm these results, and it was found that a pretreatment of DNE3 suppressed the nuclear levels of NF-*κ*B while it has no significant effect on the c-Jun and c-Fos expression, with C23 being the internal control (Figure 7(c)). The activity of NF-*κ*B is related to that of their endogenous inhibitors, IkappaB (I*κ*B), therefore Western blotting was employed to see the effect of DNE3 on I*κ*B expression, and results showed that I*κ*B protein levels were significantly increased along with the concentration of DNE3 (Figure 7(d)).

### 3.8. DNE3 Inhibit Invasion and MMP-2 and u-PA Secretion in Human Melanoma Cell Lines

**Figure 8:**
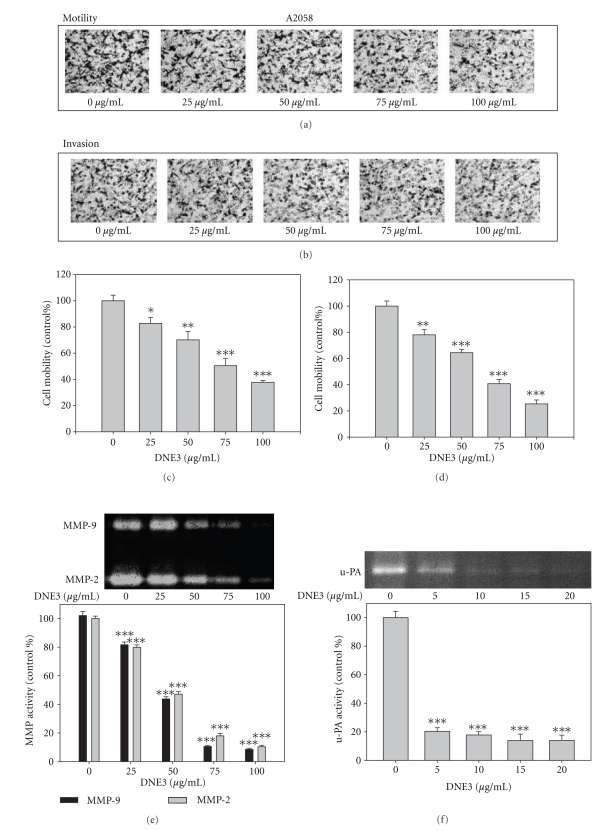
The effects of DNE3 on cell motility, invasion, and the activity of MMPs and uPA of A2058 human melanoma cells. Cells were treated with DNE3 for 24 hours and then subjected to analyze for motility ((a) and (c)) and invasion ((b) and (d)). Condition media were subjected to gelatin zymography and casein zymography to analyze the activities of MMPs (e) and u-PA (f), respectively. Data represented the mean ± SD of at least 3 independent experiments. Results were statistically evaluated by using one-way ANOVA with post hoc Dunnett's test (*P < .05; **P < .01; ***P < .001).

Similar experiments were performed, using human melanoma cells, A2058, in order to determine if the anti-invasive activity of DNE3 can generally be applicable to other types of melanoma cells. DNE3 inhibited the motility (Figures 8(a) and 8(c)) and invasion (Figures 8(b) and 8(d)) of A2058 cell lines, and DNE3 clearly inhibited the secretion of MMP-2, MMP-9 (Figure 8(e)), and u-PA (Figure 8(f)), thus indicating that DNE3 might have anti-invasive properties against a broad spectrum of melanoma cells.

## 4. Discussion and Conclusions

Chemopreventive properties have long been attributed to phenolic compounds present in the human diet and are the use of agents to delay, inhibit, or reverse tumorigenesis. These natural substances are of interest as they are potential sources of anticancer compounds with minimal debilitating toxicity and side effects [19–21]. In the present study, we demonstrated that *Dioscorea nipponica *are effective in various aspects of tumor formation in the B16F10 mouse melanoma model *in vitro. *This model was chosen on the basis of aggressive behavior and high metastatic potential of this cell line. One day after the intravenous injection of tumor cells, mice were treated with daily oral administration of the DNE3 and resulted in a significant, dose-dependent delay in lung colonization.

The metastatic process comprises a series of complicated events that can be subdivided into a number of steps involving cell motility, cell invasion, surface adhesion properties, and degradation of ECM. Thus, in the present study, we tried to investigate the antimetastatic activity of DNE3 associated with tumor thrombosis by tumor cell invasion, migration, adhesion, and proteinase expression.

Adhesive interaction of tumor cells to ECM and its invasive action into ECM are known to be fundamental events in tumor metastatic process [22]. Here, DNE3 inhibited the adhesion of B16-F10 cells to type I collagen, one of the ECM components, as well as effectively suppressed the invasion of B16-F10 cells in a concentration-dependent manner.

The secretion of extracellular proteases plays an important role in cancer cell invasion and metastasis [23]. Of these proteases, MMPs, a family of zinc-dependent ECM-degrading enzymes, are involved in invasion, migration, and angiogenesis in tumor cells. There have been reports suggesting that the expression of MMP-2 and MMP-9 correlates with the progression of various types of tumors [24]. In addition to MMPs, the serine protease u-PA converts inactive plasminogen into plasmin and therefore plays a key role to initiate a cascade of proteolytic steps accumulating in the degradation of the extracellular matrix. u-PA is found in cellular structures at the leading edge of migrating cells that are involved in cell adhesion, migration, invasion, and metastatsis [25]. MMP-2 is a proteolytic enzyme capable of degrading the structural support network for normal and malignant cells, and their expression and activity against matrix macromolecules have been shown to overbalance their endogenous inhibitors. TIMP-2 has been shown to suppress tumor growth and metastatic potential in cell and animal model systems [26]. The data presented here demonstrated that DNE3 inhibits the invasion of melanoma cancer cells and this inhibitory activity appeared to be dependent on the decreased expression of MMP-2, MMP-9, and u-PA, while that of TIMP-2 was enhanced.

The role of PI3K-Akt and MAPKs pathways in the regulation of u-PA or MMPs expressions in carcinoma cells has been well studied [23]. It has been shown that an inhibition of p-Akt may lead to a reduction in the expression of MMP-2 or u-PA as well as in the invasion of tumor cells. As shown in Figure 6, DNE3 could reduse the phosphorylation of Akt in B16-F10 cells. To make a linkage between p-Akt and protease expressions, the effects of DNE3 on several transcription factors were examined. It is well known that the activation of AP-1 and NF-*κ*B and the downstream of the PI3K-Akt pathway are associated with inflammation, cell invasion, migration, and angiogenesis [27], and that suppression of any of these transcription factors is potentially an effective means to block tumor invasion or metastasis, as well as blocking the factors that bind to these regulatory elements, and therefore represents an appropriate approach to inhibit the synthesis of MMPs or u-PA. In our studies, DNE3 was found to effectively suppress the DNA binding activity and the expression of NF-*κ*B while the protein level of I*κ*B, the endogenous inhibitor of NF-*κ*B, was gradually increased along with the concentration of DNE3.

The major drawbacks of many effective cancer chemotherapeutic agents are systemic toxicity and drug resistance. In this regard, dietary supplement as well as phytotherapeutic agents with high anticancer activity and less toxicity to normal tissues has been suggested as possible candidates for their capability to improve the efficacy of anticancer drugs [28, 29]. In the present study, HS68, human foreskin fibroblast cells, was used to demonstrate that DNE3 exerted no cytotoxicity on normal foreskin fibroblast cells. In conclusion, *Dioscorea nipponica* potently inhibits melanoma invasive and metastatic potential; therefore, they may constitute a valuable tool in the combination therapy of metastatic melanoma and in the prevention of melanoma metastases.
