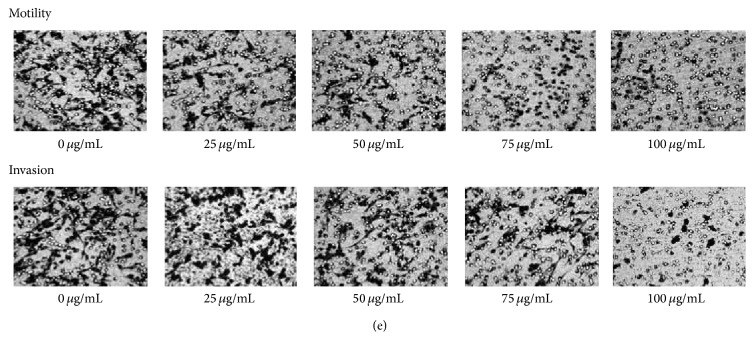# Corrigendum to “Antimetastatic Potentials of* Dioscorea nipponica* on Melanoma* In Vitro* and* In Vivo*”

**DOI:** 10.1155/2015/760692

**Published:** 2015-11-18

**Authors:** Mao-Lin Ho, Yih-Shou Hsieh, Jia-Yuh Chen, Kuo-Shuen Chen, Jia-Jing Chen, Wu-Hsien Kuo, Shu-Jiuan Lin, Pei-Ni Chen

**Affiliations:** ^1^Institute of Medicine, Chung Shan Medical University, No. 110, Section 1, Jianguo N. Road, Taichung 402, Taiwan; ^2^Department of Biochemistry, Chung Shan Medical University, No. 110, Section 1, Jianguo N. Road, Taichung 402, Taiwan; ^3^Clinical Laboratory, Chung Shan Medical University Hospital, No. 110, Section 1, Jianguo N. Road, Taichung 402, Taiwan; ^4^Department of Internal Medicine, Chung Shan Medical University Hospital, No. 110, Section 1, Jianguo N. Road, Taichung 402, Taiwan; ^5^Institute of Biochemistry and Biotechnology, Chung Shan Medical University, No. 110, Section 1, Jianguo N. Road, Taichung 402, Taiwan; ^6^Division of Gastroenterology, Department of Internal Medicine, Armed-Force Taichung General Hospital, Taichung 411, Taiwan; ^7^General Education Center, Central Taiwan University of Science and Technology, No. 11 Pu-tzu Lane, Pu-tzu Road, Taichung 406, Taiwan; ^8^Department of Pathology, Taichung Veterans General Hospital, Taichung 407, Taiwan

We have noticed a misplaced figure in our paper “Antimetastatic Potentials of* Dioscorea nipponica* on Melanoma* In Vitro* and* In Vivo*” [1]. There is an error that occurred during uploading Figure 4(e). The published microscopy image of cell invasion for treatment of* Dioscorea nipponica* ethyl acetate extracts (DNE3) on B16F10 cells is incorrect. We have attached a correct version (Figure 4(e)).

## Figures and Tables

**Figure 4:**